# Factors associated with pulmonary embolism in children with severe *Mycoplasma pneumoniae* pneumonia

**DOI:** 10.3389/fimmu.2026.1900602

**Published:** 2026-10-09

**Authors:** Xuelian Chang, Liangkai Zhang, Zhongxiao Zhang, Xiaodi Tang, Mengjiao Zhao, Qian Li, Linlin Han, Jing Ma, Xiuli Yan

**Affiliations:** Respiratory and Interventional Radiology, Children’s Hospital Affiliated to Shandong University/Jinan Children’s Hospital, Jinan, China

**Keywords:** children, D-dimer, mycoplasma pneumoniae, pneumonia, mycoplasma, pulmonary embolism

## Abstract

**Background:**

To investigate the clinical characteristics and factors for pulmonary embolism (PE) in children with severe *Mycoplasma pneumoniae* pneumonia (SMPP).

**Methods:**

This retrospective study included pediatric patients with SMPP hospitalized at the Affiliated Children’s Hospital of Shandong University between October 2019 and March 2025. They were divided into the PE and control groups based on the presence/absence of PE. Multivariable logistic regression analysis was performed to identify factors independently associated with PE in children with SMPP. The area under the curve (AUC) of the receiver operating characteristic (ROC) curve was calculated to evaluate the discriminative ability of the identified factors.

**Results:**

Among the 199 children with SMPP included in this study, 100 were diagnosed with PE and 99 served as controls. The mean age was 6.73 ± 1.79 years in the PE group and 6.57 ± 1.70 years in the control group. The mean interval from symptom onset to PE diagnosis was 17 days. The multivariable logistic regression analysis indicated that duration of fever (OR = 1.122, 95%CI: 1.040-1.211, P = 0.003), extrapulmonary symptoms (OR = 3.479, 95%CI: 1.853-6.535, P<0.001), NEU (OR = 1.355, 95%CI: 1.131-1.624, P = 0.001), ESR (OR = 1.024, 95%CI: 1.003-1.047, P = 0.028), APTT (OR = 0.926, 95%CI: 0.866-0.990, P = 0.025), D-dimer level (OR = 1.210, 95%CI: 1.111-1.319, P<0.001), and cardiovascular complications (OR = 9.500, 95%CI: 1.759-51.316, P = 0.009) were independently associated with PE in children with SMPP. D-dimer showed the largest AUC (AUC = 0.806), with a sensitivity of 75% and a specificity of 74%.

**Conclusion:**

Early clinical signs of pulmonary embolism (PE) in children with SMPP include nonspecific prolonged fever, elevated inflammatory markers, increased D-dimer, and cardiovascular complications.

## Introduction

*Mycoplasma pneumoniae* pneumonia (MPP) is a common form of community-acquired pneumonia in children, predominantly affecting those aged 5 years and older (1). Its incidence has shown a steady upward trend in recent years, especially due to the increasing prevalence of macrolide-resistant *M. pneumoniae*, leading to severe and critical cases (2, 3). In addition to respiratory manifestations, MPP can lead to various complications, including pulmonary complications such as plastic bronchitis, pleural effusion, and necrotizing pneumonia, as well as extrapulmonary complications involving the cardiovascular, nervous, hematologic, and mucocutaneous systems (1–3).

Pulmonary embolism (PE) is one of the most severe complications of MPP, significantly affecting prognosis, and is also included as a diagnostic criterion for severe MPP (4, 5). PE refers to a group of clinical conditions caused by obstruction of the pulmonary artery or its branches by emboli, most commonly pulmonary thromboembolism (PTE) (6, 7). Although PE is relatively rare in children, it is potentially life-threatening and its incidence has increased in recent years. In China, the reported incidence is 0.14-0.9 per 100, 000 children, while among hospitalized children, the incidence reaches 8.6-92.0 per 100, 000 (8). Despite its relatively low incidence, PE is associated with substantial morbidity and mortality in pediatric patients, imposing a considerable clinical and healthcare burden.

The risk factors for pediatric PE include conditions that promote blood stasis, endothelial injury, or a hypercoagulable state. These factors may be primary, such as genetic predisposition, or secondary, including infection, congenital heart disease, nephrotic syndrome, indwelling central venous catheters, parenteral nutrition, prolonged immobilization, and malignancy (9). Among these, infection—particularly *M. pneumoniae* infection—is considered one of the most common causes in China (10).

Infection is a well-recognized risk factor for PE, and current evidence indicates that *M. pneumoniae* infection is one of the most common infectious causes of PE (11). However, the classic triad of chest pain, hemoptysis, and dyspnea is uncommon in pediatric PE. Instead, its clinical manifestations often overlap with those of severe pneumonia, making early recognition challenging (8). Consequently, PE may be easily overlooked, resulting in delayed diagnosis and treatment, which can lead to poor outcomes and even life-threatening complications (8). Therefore, identifying children with MPP who are at higher risk of developing PE remains an important clinical challenge. This study aimed to analyze the clinical characteristics of children with severe MPP and the factors associated with PE. The results could help enhance clinicians’ ability to recognize and intervene early and potentially improve patient outcomes.

## Materials and methods

### Study design and population

This retrospective study included pediatric patients with severe MPP complicated by PE hospitalized at the Affiliated Children’s Hospital of Shandong University between October 2019 and March 2025. This study was approved by the ethics committee of the Affiliated Children’s Hospital of Shandong University (approval No. SDFE-IRB/P-2022055). Given the study’s retrospective nature, the requirement for individual informed consent was waived. The study was conducted in accordance with the ethical principles of the Declaration of Helsinki.

The patients with severe MPP and PE were assigned to the PE group. During the same period, children diagnosed with severe MPP who underwent CTPA or CTA but had negative imaging results for PE were included as the control group. At our center, chest CT is commonly performed early in children with MPP to evaluate the extent of pulmonary involvement, which is one of the diagnostic considerations for severe MPP. Contrast-enhanced chest CT is subsequently performed in some patients to assess pulmonary ischemia or necrosis, guide prognosis evaluation and follow-up planning, and evaluate severe complications such as PE or cardiac embolism. The inclusion criteria for the PE group were (1) age <18 years (2), fulfillment of the diagnostic criteria for MPP according to the Guidelines for the Diagnosis and Treatment of Mycoplasma pneumoniae Pneumonia in Children (2023 Edition) (4), and 3) evidence of PE on CTPA or CT angiography (CTA), defined as intraluminal filling defects of the pulmonary artery, or absence or reduction of distal pulmonary arterial branches. The inclusion criteria for the control group were (1) children diagnosed with severe MPP, and (2) PE was excluded by CTPA or CTA. The exclusion criteria were (1) history of PE (2), PE caused by other etiologies, including tumor embolism, air embolism, or embolism associated with hereditary disorders, nephrotic syndrome, congenital heart disease, central venous catheterization, surgery, or other factors, or (3) incomplete clinical data.

MPP was diagnosed based on the presence of clinical manifestations and etiological evidence, including (1) clinical symptoms of fever and cough (2), infiltrative changes on chest radiography or chest computed tomography (CT) (3), serological evidence defined as a single serum *M. pneumoniae* antibody titer ≥1:160 using the particle agglutination (PA) method, or a ≥4-fold increase in paired serum antibody titers during the disease course, and (4) positive detection of MP-DNA or MP-RNA (4).

### Routine management

All patients received routine treatment after admission, including anti-infective therapy, anti-inflammatory therapy, and expectorant treatment. Anticoagulation therapy was administered to patients with confirmed thrombosis as clinically indicated. For anti-infective therapy, macrolide antibiotics were selected as first-line treatment in the early stage, including azithromycin (10 mg/kg/day, once daily for 7 days, with a second course initiated after an interval of 3–4 days) and erythromycin (30–45 mg/kg/day for 10–14 days). With the increasing incidence of severe MPP, poor clinical response to macrolide therapy after 72 hours, positive results for macrolide resistance gene testing, and exclusion of concomitant infections, second-line antibiotics were gradually initiated. These included levofloxacin (6 months to 5 years: 8–10 mg/kg per dose, every 12 h; 5–16 years: 8–10 mg/kg per dose, once daily; treatment duration 7–14 days) and minocycline (loading dose 4 mg/kg per dose, followed by 2 mg/kg per dose every 12 h after a 12-h interval; treatment duration 10 days).

### Treatments in the PE group

Among the 100 patients in the PE group, anti-infective regimens included azithromycin alone in 54, azithromycin replaced with levofloxacin in 17, levofloxacin alone in 25, erythromycin in 1, minocycline in 1, ceftazidime in 1, and meropenem combined with linezolid in 1. For anticoagulation therapy, intravenous unfractionated heparin was administered during the early study period in 44 patients: 9 received 50–100 IU/kg per dose every 12 h, while 35 received 80 IU/kg followed by 18 IU/kg/h. APTT was dynamically monitored every 4 hours to assess therapeutic anticoagulation levels. Given the lower risk of bleeding and the lack of requirement for coagulation monitoring, Low-molecular-weight heparin (LMWH) gradually replaced unfractionated heparin in the later study period. Fifty-six patients received subcutaneous LMWH at 100 IU/kg every 12 h. Long-term anticoagulation therapy was subsequently continued with oral warfarin (0.1-0.2 mg/kg, maximum dose 5 mg) or rivaroxaban (dose adjusted according to body weight). Thoracentesis was performed in 26 patients in the embolism group. Eight patients were admitted to the intensive care unit due to respiratory failure. Flexible bronchoscopy with bronchoalveolar lavage (FBAL) was performed in 97 patients in the PE group.

### Data collection

Within 24 hours of admission, all patients underwent etiological testing for *M. pneumoniae*, including detection of *M. pneumoniae*-specific IgM antibodies and *M. pneumoniae* nucleic acid testing. Additional pathogen testing, including macrolide resistance gene detection, pathogen capture-based metagenomic sequencing, sputum culture, and blood culture, was performed according to clinical indications.

Clinical data were collected from all included patients (1): demographic and baseline characteristics: sex, age, body weight, length of hospital stay, history of venous thromboembolism, and pre-existing cardiovascular diseases (2), clinical manifestations: body temperature, duration of fever, cough, chest tightness, chest pain, hemoptysis, shortness of breath, dyspnea, extrapulmonary symptoms, intensive care unit (ICU) admission, and extrapulmonary complications. Extrapulmonary symptoms referred to non-respiratory symptoms present at admission, including abdominal pain, headache, lower limb swelling, lower limb pain, and limb motor dysfunction. Extrapulmonary complications referred to non-respiratory organ-system involvement confirmed by clinical evaluation or auxiliary examinations and were recorded separately by system (3). laboratory findings: complete blood count, C-reactive protein (CRP), erythrocyte sedimentation rate (ESR), procalcitonin, ferritin (FER), blood biochemical indices, coagulation parameters, immunoglobulin A, G, and M levels, T-lymphocyte subsets, and cytokine profiles (4), imaging and auxiliary examinations: chest imaging, cranial imaging, lung ultrasonography, echocardiography, ultrasonography of peripheral and abdominal vessels, and electrocardiography (5), distribution of concomitant pathogenic infections (6), treatment in the PE group: anti-infective therapy, anticoagulation therapy, anti-inflammatory treatment, mechanical ventilation, bronchoscopic intervention, catheter-directed local thrombolytic therapy, and thoracentesis, and (7) follow-up and prognosis.

### Statistical analysis

Statistical analyses were performed using SPSS 24.0 (IBM Corp., Armonk, NY, USA). The normality of continuous variables was assessed using the Shapiro–Wilk test. Continuous variables with normal distributions were reported as means ± standard deviation (SD) and compared between groups using the independent-samples t-test. Non-normally distributed continuous variables were presented as medians and interquartile ranges [P50 (P25, P75)] and compared using the Mann-Whitney U test. Categorical variables were expressed as frequencies and percentages and compared using the chi-square test. Before regression modeling, 17 candidate variables were prespecified based on clinical relevance, data completeness, and avoidance of potentially redundant measures. Each candidate variable was evaluated using univariable logistic regression. The 15 variables with P < 0.05 were entered into the multivariable logistic regression model, and seven variables with P < 0.05 in that model were retained in the final association model. Multicollinearity among these seven variables was assessed using variance inflation factors (VIFs). As a penalized sensitivity analysis, LASSO logistic regression was subsequently applied to the seven variables retained in the multivariable model using eightfold cross-validation and the one-standard-error criterion (λ1se). The final model was internally validated using 1, 000 bootstrap resamples, and the apparent and optimism-corrected AUCs were calculated. Calibration was assessed using the Hosmer–Lemeshow goodness-of-fit test and a calibration plot. Receiver operating characteristic (ROC) curve analysis was used to evaluate the discriminative ability of study indicators for PE by calculating the area under the curve (AUC) and to determine the optimal cutoff values. All statistical tests were two-sided, and P values < 0.05 were considered statistically significant.

## Results

### Characteristics and clinical manifestations of the patients

A total of 199 children with severe MPP were included in the analysis, comprising 100 patients in the PE group and 99 patients in the control group. The overall study population had a mean age of approximately 6.6 years, and 62% were male, with no significant differences in age or sex distribution between the two groups (both P>0.05). Among the baseline characteristics, body weight (27.62 ± 10.76 vs. 24.60 ± 7.35, P = 0.022) and length of hospital stay (14.54 ± 4.96 days vs. 11.34 ± 2.99 days, P<0.001) were significantly higher in the PE group compared with the control group. Regarding clinical manifestations, children in the PE group had a longer fever duration, higher peak body temperature, higher heart rate, higher incidence of hemoptysis, and more frequent extrapulmonary symptoms than those in the control group (all P<0.05). In terms of imaging findings, pulmonary consolidation involving ≥2/3 of the lung and necrotizing pneumonia were significantly more common in the PE group (both P < 0.01). In contrast, no significant differences were observed between the two groups in most respiratory symptoms (including fever, cough, chest pain, chest distress, dyspnea, and shortness of breath), other imaging findings, bronchoscopy findings, or several extrapulmonary complications (all P > 0.05) (**Table 1**).

**Table 1:** General information, clinical manifestations, and imaging findings.

Variables	PE (n=100)	Control (n=99)	P
Age (years)	6.73 ± 1.79	6.57 ± 1.70	0.515
Male, n, %	62, 62.00%	62, 62.63%	0.927
Weight (kg)	27.62 ± 10.76	24.60 ± 7.35	0.022
Length of hospital stay (days)	14.54 ± 4.96	11.34 ± 2.99	<0.001
Symptoms			
Fever, n, %	99, 99.00%	98, 98.99%	0.994
Fever duration (days)	10 (7, 14)	8 (7, 10)	0.004
Highest body temperature (°C)	40.00 (39.65, 40.20)	39.90 (39.30, 40.10)	0.040
Heart rate (bpm)	112.0 (102.5, 120.0)	105.0 (98.0, 116.0)	0.013
Cough, n, %	100, 100.00%	99, 100.00%	
Chest pain, n, %	11, 11.00%	9, 9.09%	0.654
Chest distress, n, %	4, 4.00%	1, 1.01%	0.178
Hemoptysis, n, %	5, 5.00%	0, 0.00%	0.024
Dyspnea, n, %	6, 6.00%	1, 1.01%	0.056
Shortness of breath, n, %	13, 13.00%	6, 6.06%	0.096
Extrapulmonary symptoms, n, %	58, 58.00%	29, 29.29%	<0.001
Imaging, n, %			
Pulmonary consolidation involving ≥2/3 of the lung	53, 53.00%	25, 25.25%	<0.001
Isolated pulmonary consolidation	9, 9.00%	34, 34.34%	<0.001
Pulmonary consolidation or atelectasis	35, 35.00%	23, 23.23%	0.068
Isolated atelectasis	56, 56.00%	42, 42.42%	0.055
Pleural effusion	58, 58.00%	48, 48.48%	0.179
Pleural thickening	2, 2.00%	2, 2.02%	0.992
Necrotizing pneumonia	46, 46.00%	26, 26.26%	0.006
Ischemia	53, 53.00%	64, 64.63%	0.095
Pneumothorax	1, 1.00%	0, 0.00%	0.319
Lung abscess	1, 1.00%	0, 0.00%	0.319
Bronchoscopy findings, n, %			
Endobronchitis	59, 59.00%	67, 67.68%	0.204
Mucus plug	57, 57.00%	51, 51.52%	0.437
Plastic bronchitis	40, 40.00%	46, 46.46%	0.357
Bronchial stenosis	26, 26.00%	29, 29.29%	0.604
Bronchiectasis	4, 4.00%	6, 6.06%	0.506
Bronchoscopy procedure	97, 97.00%	97, 97.98%	0.063
Extrapulmonary complications, n, %			
Nervous system	5, 5.00%	2, 2.02%	0.254
Circulatory system	42, 42.00%	14, 14.14%	<0.001
Digestive system	53, 53.00%	21, 21.21%	0.001
Blood system	6, 6.00%	7, 7.07%	0.760
Cutaneous mucosal system	1, 1.00%	7, 7.07%	0.029
Urinary system	4, 4.00%	0, 0.00%	0.044
Co-pathogen infection	40, 40.00%	74, 74.75%	<0.001
Bacteria	10, 10.00%	33, 33.33%	
Virus	26, 26.00%	41, 41.41%	
Fungi	4, 4.00%	0, 0.00%	
Time from symptom onset to hospital admission (days)	10 (8, 14)	9 (7, 11)	0.003

PE, pulmonary embolism.

The mean interval from symptom onset to PE diagnosis was 17 days. CTPA or CTA in the 100 patients with PE demonstrated single or multiple complete or partial intraluminal filling defects in the pulmonary arteries or pulmonary veins (**Figure 1**, **Supplementary Table S1).** Notably, only 20% of PEs were located in the same lung region as the site of infection.

**Figure 1:**
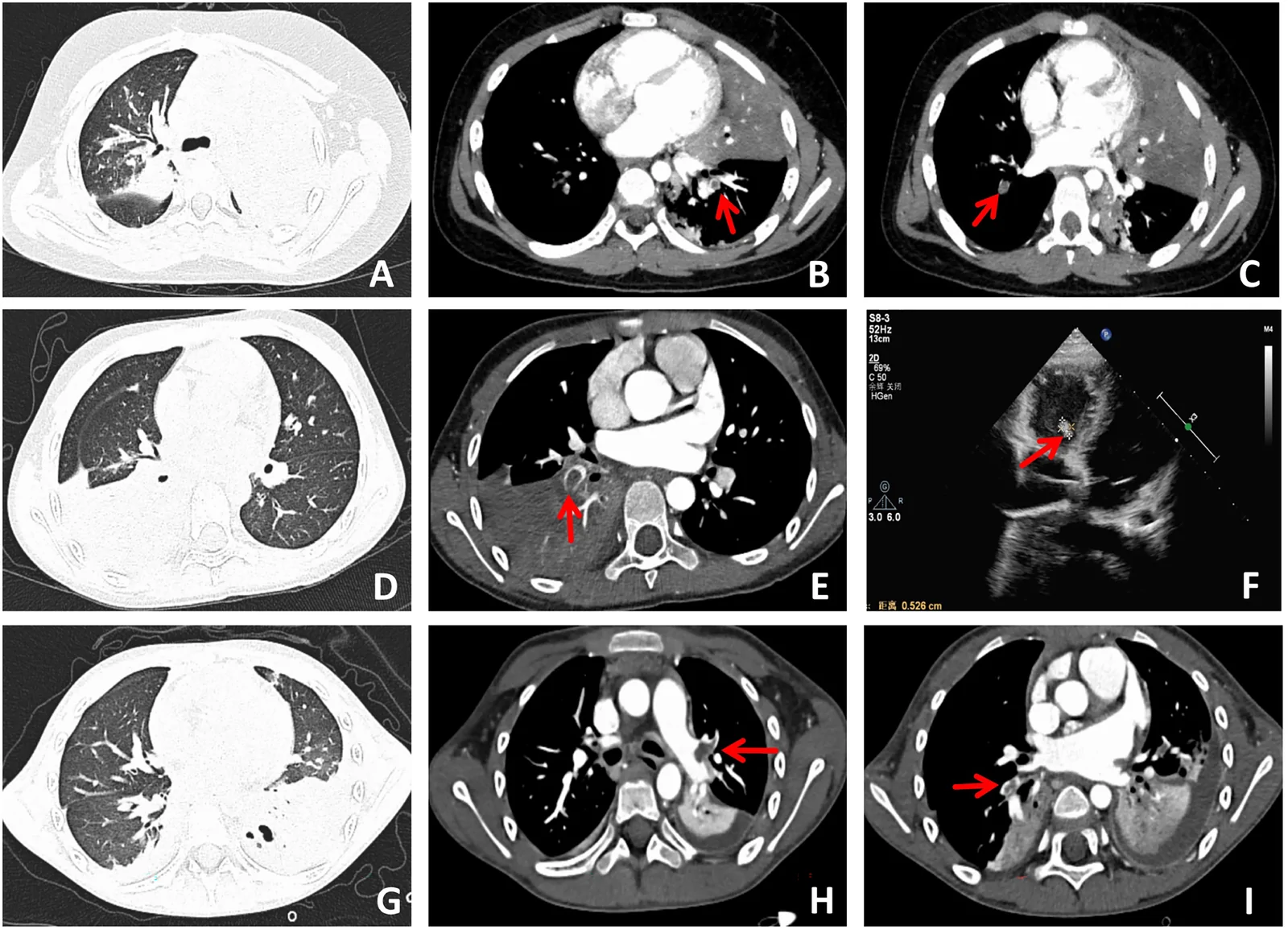
Imaging manifestations of children with pulmonary embolism on computed tomography pulmonary angiography (CTPA) or computed tomography angiography (CTA). Patient 1 **(A–C)** Left upper lobe of the lung is atelectatic with partial ischemic and necrotic changes. Partial pulmonary artery branches in both lower lungs show filling defects. Patient 2 **(D, E)** Partial consolidation and atelectasis in the lower lobe of the right lung, with partial ischemia and necrosis; right pleural effusion; filling defects are observed in the right lower pulmonary artery and its branches, as well as in some branches of the left lower pulmonary artery. **(F)** Cardiac ultrasound shows a medium-echo mass approximately 0.8×0.7×0.5 cm in size attached to the anterior chordae tendineae of the tricuspid valve in the right ventricle. Patient 3 **(G–I)** Pneumonia with multiple consolidations and atelectasis. Partial ischemic necrosis of the lower lobe of the left lung, accompanied by formation of pulmonary air cysts; Bilateral pleural effusion. Multiple low-density filling defects are seen in the pulmonary arteries, the largest located at the opening of the left pulmonary artery branch, measuring approximately 12.8×7.1 mm.

### Laboratory findings in the two groups

Compared with the control group, children in the PE group had higher levels of white blood cell (WBC) count, neutrophil count (NEU), neutrophil percentage (NEUT%), CRP, ESR, procalcitonin (PCT), FER, creatine kinase-MB isoenzyme (CK-MB), lactate dehydrogenase (LDH), α-hydroxybutyrate dehydrogenase (α-HBDH), aspartate aminotransferase (AST), prothrombin time (PT), thrombin time (TT), and D-dimer (all P<0.05). In contrast, red blood cell (RBC) count, lymphocyte percentage (LYM%), alanine aminotransferase (ALT), albumin-to-globulin ratio (A/G), and activated partial thromboplastin time (APTT) were lower in the PE group than in the control group (all P<0.05) (**Table 2**).

**Table 2:** Laboratory variables between the groups.

Variables	Reference value	PE (n=100)	Control (n=99)	P
WBC	4.4-11.9×10^9^/L	11.48 ± 4.05	9.97 ± 3.62	0.007
RBC	4-5.5×10^12^/L	4.19 ± 0.58	4.44 ± 0.55	0.003
PLT	188-472×10^9^/L	299.25 ± 117.15	330.06 ± 129.33	0.081
NEU	1.2-7×10^9^/L	8.67 ± 3.38	6.73 ± 2.69	<0.001
LC	1.8-6.3×10^9^/L	2.04 ± 1.38	2.31 ± 1.33	0.184
NEUT%	22-65%	74.40 ± 12.69	67.63 ± 12.25	<0.001
LYM%	23-69%	17.64 ± 9.27	23.81 ± 11.15	<0.001
CRP	0–10 mg/L	39.59 ± 37.76	25.63 ± 28.82	0.004
ESR	0–20 mm/h	41.64 ± 24.51	32.32 ± 17.55	0.003
PCT	<0.50 ng/ml	0.35 ± 0.47	0.21 ± 0.26	0.017
FER	30-400 µg/L	387.00 (244.00, 1177.00)	159.9 (116.98, 320.50)	0.001
CK	44.11-211.69 U/L	78.00 (31.00, 158.00))	57.50 (38.75, 195.75)	0.984
CKMB	0-5.00 ng/ml	2.19 (0.90, 6.41)	1.21 (1.00, 1.53)	<0.001
LDH	109–245 U/L	584.59 ± 360.77	424.70 ± 246.94	<0.001
α-HBDH	74–200 U/L	401.98 ± 207.15	290.25 ± 146.97	<0.001
ALT	7–30 U/L	28.00 (13.00, 119.00)	34.00(14.50, 52.50)	<0.001
AST	14–44 U/L	43.00 (31.00, 66.00)	29.00 (21.00, 39.50)	0.01
Albumin	39.0-54.0 g/L	34.57 ± 4.24	35.73 ± 4.56	0.071
Globulin	20–30 g/L	27.34 ± 6.05	25.92 ± 4.51	0.076
A/G	1.2-2.3	1.34 ± 0.34	1.44 ± 0.26	0.042
PT	10.0-14.0 s	12.93 ± 1.68	12.47 ± 1.09	0.022
INR	0.8-1.25	1.10 ± 0.14	1.07 ± 0.10	0.079
APTT	22.0-38.0 s	26.61 ± 5.83	29.20 ± 5.25	0.001
TT	14.0-21.0 s	16.39 ± 1.75	15.77 ± 1.89	0.018
Fg	2.0-4.0 g/L	3.61 ± 1.24	3.65 ± 1.04	0.813
D-dimer	0-0.50 mg/L	8.76 ± 7.70	2.97 ± 4.47	<0.001
IL-6	0-16.6 pg/ml	47.72 (17.55, 184.77)	26.84 (11.68, 49.81)	0.125
IL-10	0-4.9 pg/ml	10.58 (6.68, 22.75)	11.16 (7.18, 14.94)	0.940
IL-2	0-9.8 pg/ml	2.03 ± 1.69	1.79 ± 1.11	0.645
IL-4	0–3 pg/ml	2.09 (1.16, 4.69)	1.94 (1.26, 2.53)	0.783
IgA	0.46-2.5 g/L	1.43 (0.98, 3.28)	1.49 (1.20, 1.89)	0.204
IgM	0.29-1.41 g/L	1.90 (1.65, 2.90)	1.32 (0.96, 2.35)	0.053
IgG	6.3–15 g/L	9.62 (8.14, 13.06)	9.38 (7.93, 11.50)	0.365
Total T cell absolute count	53.37-71.91	67.70 ± 12.03	68.41 ± 10.19	0.681
Relative count of helper/inducer T cells	26.19-45.48	33.78 ± 9.83	35.47 ± 8.85	0.246
Relative count of suppressive/cytotoxic T cells	16.29-29.88	32.72 ± 9.49	31.26 ± 9.74	0.329
Ratio	1.05-2.53	1.08 ± 0.41	1.27 ± 0.46	0.004

PE, pulmonary embolism; WBC, white blood cell; RBC, red blood cell; PLT, platelets; NEU, neutrophil count; LC, lymphocyte count; CRP, C-reactive protein; ESR, erythrocyte sedimentation rate; PCT, procalcitonin; FER, ferritin; CK, creatine kinase; CKMB, creatine kinase-MB; LDH, lactate dehydrogenase; α-HBDH, α-hydroxybutyrate dehydrogenase; ALT, alanine transaminase; AST, aspartate transaminase; PT, prothrombin time; INR, international normalized ratio; APTT, activated partial thromboplastin time; TT, thrombin time; Fg, fibrinogen; IL-6, interleukin-6; IL-10, interleukin-10; IL-2, interleukin-2; IL-4, interleukin-4; IgA, immunoglobulin A; IgM, immunoglobulin M; IgG, immunoglobulin G. P values were calculated using the independent-samples t-test or Mann-Whitney U test according to data distribution.

### Concomitant pathogenic infections

All patients underwent etiological testing after admission and were confirmed to be positive for *M. pneumoniae*. Among them, 37 patients underwent macrolide resistance testing, which yielded positive results. Point mutations A2063G and A2064G in the V23S rRNA region were identified, with A2063G being the predominant mutation. Because only 37 of 199 patients underwent macrolide resistance testing, these data were considered descriptive only and were not used to assess the association between macrolide resistance and PE. A statistically significant difference was observed between the PE and control groups in the distribution of concomitant pathogenic infections (P<0.001). The PE group had mixed infections involving bacteria, viruses, and fungi, whereas the control group had concomitant infections limited to bacteria and viruses (**Table 1**, **Figure 2**).

**Figure 2:**
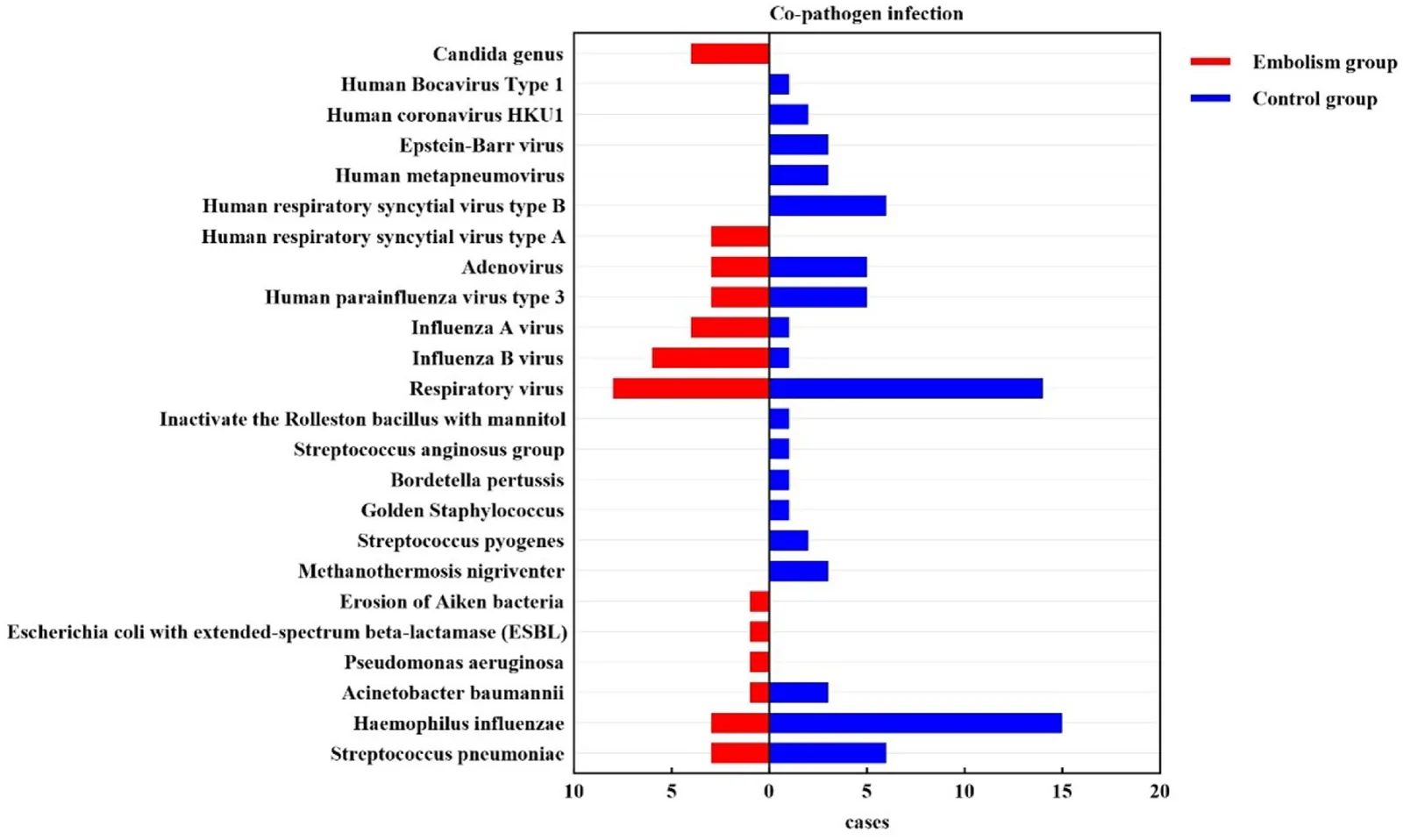
Co-pathogen infection.

### Factors independently associated with PE

Before regression modeling, 17 candidate variables were prespecified: fever duration, highest body temperature, extrapulmonary symptoms, neutrophil count, CRP, ESR, FER, LDH, α-HBDH, PT, APTT, TT, D-dimer, pulmonary consolidation involving ≥2/3 of the lung, necrotizing pneumonia, cardiovascular complications, and digestive system complications. Of these, 15 variables were associated with PE in univariable logistic regression (P < 0.05) and were entered into the multivariable analysis. The final multivariable model retained seven factors independently associated with PE: fever duration (OR = 1.122, 95% CI: 1.040–1.211, P = 0.003), extrapulmonary symptoms (OR = 3.479, 95% CI: 1.853–6.535, P < 0.001), neutrophil count (OR = 1.355, 95% CI: 1.131–1.624, P = 0.001), ESR (OR = 1.024, 95% CI: 1.003–1.047, P = 0.028), APTT (OR = 0.926, 95% CI: 0.866–0.990, P = 0.025), D-dimer (OR = 1.210, 95% CI: 1.111–1.319, P < 0.001), and cardiovascular complications (OR = 9.500, 95% CI: 1.759–51.316, P = 0.009) (**Table 3**).

**Table 3:** Logistic regression analysis of children with severe pneumonia and *Mycoplasma pneumoniae* complicated with pulmonary embolism.

Variables	Univariate logistic regression			Multivariate logistic regression			
	OR	95%CI	P	OR	95%CI	P
Symptoms								
Fever duration (days)	1.106	1.028, 1.190	0.007	1.122	1.040, 1.211	0.003
Highest body temperature	0.989	0.856, 1.142	0.877			
Extrapulmonary symptoms	3.333	1.853, 5.998	<0.001	3.479	1.853, 6.535	<0.001
Inflammatory indicators								
NEU	1.239	1.113, 1.378	<0.001	1.355	1.131, 1.624	0.001
CRP	1.013	1.004, 1.022	0.006			
ESR	1.021	1.007, 1.035	0.004	1.024	1.003, 1.047	0.028
FER	1.000	1.000, 1.001	0.160			
LDH	1.002	1.001, 1.004	0.001			
α-HBDH	1.004	1.002, 1.007	<0.001			
Coagulation indicators								
PT	1.300	1.029, 1.643	0.028			
APTT	0.914	0.863, 0.968	0.002	0.926	0.866-0.990	0.025
TT	1.226	1.030-1.458	0.022			
D-dimer	1.245	1.142, 1.357	<0.001	1.210	1.111, 1.319	<0.001
Imaging								
Pulmonary consolidation involving ≥2/3 of the lung	3.338	1.832, 6.081	<0.001			
Necrotizing pneumonia	2.297	1.265, 4.170	0.006			
Extrapulmonary complications								
Cardiovascular system	4.397	2.203, 8.773	<0.001	9.500	1.759, 51.316	0.009
Digestive system	2.918	1.565, 5.441	0.001			

OR, odds ratio; CI, confidence interval; NEU, neutrophil count; CRP, C-reactive protein; ESR, erythrocyte sedimentation rate; FER, ferritin; LDH, lactate dehydrogenase; α-HBDH, α-hydroxybutyrate dehydrogenase; PT, prothrombin time; APTT, activated partial thromboplastin time; TT, thrombin time.

As a penalized sensitivity analysis, LASSO logistic regression was applied to the seven variables retained in the multivariable model. At λ1se = 0.0477, all seven variables had nonzero coefficients: fever duration, 0.0089; extrapulmonary symptoms, 0.4652; neutrophil count, 0.0937; ESR, 0.0109; APTT, −0.0832; D-dimer, 0.0880; and cardiovascular complications, 0.6175 (**Supplementary Figure S1**). Because LASSO was applied only to the seven variables retained in the multivariable model, this analysis was interpreted as a penalized sensitivity analysis rather than as an independent variable-selection procedure.

The VIFs for fever duration, extrapulmonary symptoms, neutrophil count, ESR, APTT, D-dimer, and cardiovascular complications were 1.106, 2.315, 1.153, 1.097, 1.120, 1.145, and 2.191, respectively. All VIFs were below 2.5, indicating no evidence of problematic multicollinearity. Bootstrap internal validation with 1, 000 resamples yielded an apparent AUC of 0.886 and an optimism-corrected AUC of 0.865. The Hosmer–Lemeshow test showed no evidence of lack of fit (χ² = 5.676, P = 0.683), and the apparent and bootstrap bias-corrected calibration curves were close to the ideal line (**Supplementary Figure S2**).

ROC curve analysis was performed to evaluate the discriminative ability of D-dimer level, NEU, and ESR for PE in severe MPP. D-dimer showed the largest AUC (AUC = 0.806), with a sensitivity of 75.0%, specificity of 74.0%, positive predictive value of 74.5%, and negative predictive value of 74.6%. Based on the maximum Youden index, the optimal cutoff values were 3.2 mg/L for D-dimer, 8.83×10^9^/L for NEU, and 57.5 mm/h for ESR (**Supplementary Table S2**, **Figure 3**).

**Figure 3:**
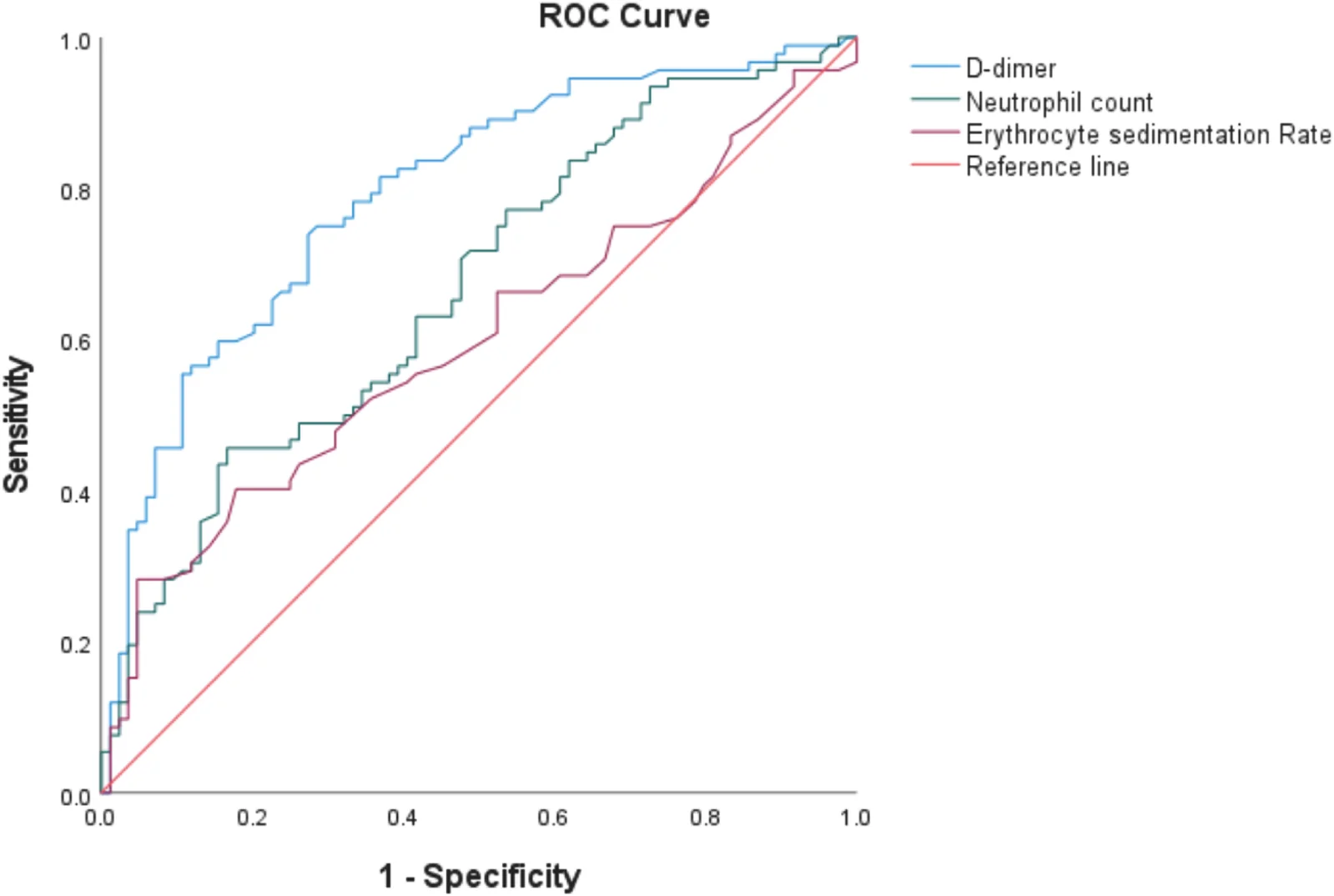
Receiver operating characteristic (ROC) analysis.

### Follow-up and prognosis

No deaths occurred among the included patients. The children in the PE group were followed for 6 months to 1 year after discharge, and no thrombosis recurrence was observed. Follow-up CTPA showed that, in all 100 children with PE, the mean time from PE diagnosis to complete disappearance of intravascular thrombi was 49.22 days (range, 7–195 days). Among the seven patients with cardiac embolism, five showed complete resolution of intracardiac thrombi at 1 month. Of the remaining two patients, one still had residual thrombus in the right ventricle at 2 months of follow-up, and the other had persistent thrombus in the right atrium at 1.25 years, although the thrombus was markedly reduced in size. After PE was confirmed by contrast-enhanced chest CT, all patients in the PE group underwent vascular ultrasonography of the extremities to evaluate peripheral vascular thrombosis. Seven patients had concomitant peripheral vascular thrombosis of the extremities, involving the upper or lower limbs, and the mean time to complete thrombus resolution was 10.33 days. Two patients with significant neurological sequelae following cerebral embolism received rehabilitation therapy, and both motor and language functions showed improvement compared with baseline.

## Discussion

This retrospective study investigated the clinical characteristics and factors associated with PE in children with severe MPP. The results suggested that the early clinical manifestations of PE in children with SMPP are often nonspecific. Seven factors were independently associated with PE: prolonged fever, extrapulmonary symptoms, increased neutrophil count, elevated ESR, shortened APTT, increased D-dimer level, and cardiovascular complications. These factors reflect three clinically relevant domains: sustained systemic inflammation, activation of coagulation and fibrinolysis, and multisystem or cardiovascular involvement.

The rising incidence of PE is thought to be associated with increased clinical awareness, advances in noninvasive diagnostic techniques, and the more frequent use of central venous catheters (12). Previous studies have reported a bimodal age distribution of pediatric PE, primarily affecting infants and adolescents (10). In contrast, the present study showed that PE in severe MPP mainly occurred in school-aged children, which may be related to the epidemiological characteristics of M. *pneumoniae* infection. Although males predominated, no significant sex difference was observed.

The main clinical manifestations of MPP are cough and fever. In this study, nearly all patients presented with fever, and longer fever duration was independently associated with PE. Prolonged fever may reflect an excessive inflammatory response following *M. pneumoniae* infection, leading to endothelial injury and promoting thrombogenesis (13). Consistent with previous reports, there was a delay of approximately two weeks between initial symptoms and PE diagnosis (14, 15), likely due to the nonspecific presentation and overlap with pulmonary complications of MPP. Extrapulmonary symptoms were also independently associated with PE, suggesting that multisystem involvement may indicate a stronger systemic inflammatory response. In addition, cardiovascular complications showed a significant association with PE, which may be explained by systemic inflammatory responses leading to vascular injury, ischemia, and thrombosis. Therefore, when children with severe MPP present with prolonged fever, extrapulmonary symptoms, cardiovascular involvement, or clinical deterioration, clinicians should consider the possibility of PE.

The mechanisms underlying PE secondary to MPP remain unclear but are likely related to infection-induced hypercoagulability. M. pneumoniae may damage vascular endothelial cells and activate coagulation pathways (16). From an immunological perspective, M. pneumoniae infection may also promote thrombosis through immunothrombosis, in which innate immune activation, endothelial injury, and coagulation pathways interact. Neutrophil activation and neutrophil extracellular trap formation, complement activation, and increased tissue factor expression may amplify thrombus formation (17). This mechanism may help explain the association of PE with prolonged fever, elevated inflammatory markers, increased neutrophil count, and D-dimer elevation in the present study. Anticardiolipin antibodies can induce a hypercoagulable state, and *M. pneumoniae* infection has been shown to stimulate the production of anticardiolipin antibodies, which may play a role in the pathogenesis of PE (18). Song et al. (14) reported that transient autoimmune abnormalities occur during thrombus formation in children with MPP complicated by PE, and that these immune responses contribute to thrombogenesis.

D-dimer was independently associated with PE and demonstrated good discriminative ability for distinguishing children with PE from those without PE. Based on ROC curve analysis and the maximum Youden index, a D-dimer cutoff value of 3.2 mg/L was associated with a higher likelihood of PE, with a sensitivity of 75.0%, specificity of 74.0%, positive predictive value of 74.5%, and negative predictive value of 74.6%. Although D-dimer is highly sensitive, its specificity is limited in the context of infection (19, 20). MPP complicated by PE is often accompanied by elevated plasma D-dimer levels (21). The cutoff value in the present study differs from those reported in previous studies, which may be related to differences in disease severity, timing of PE development, and timing of D-dimer measurement. In this study, D-dimer was measured within 24 hours after admission to our center; however, the duration of illness before admission varied among patients, and some children may not yet have developed PE at the time of blood sampling whereas others may already have had PE. Therefore, this cutoff should be interpreted as an admission-based warning threshold for identifying children with SMPP at increased risk of PE, rather than a definitive diagnostic threshold. APTT was also independently associated with PE. A shortened APTT reflects accelerated blood coagulation and may indicate a hypercoagulable state (22). Together, increased D-dimer and shortened APTT suggest that coagulation activation and fibrinolysis are important features of PE in children with SMPP.

Inflammatory markers were significantly elevated in the PE group. Increased NEU and ESR were independently associated with PE, supporting the role of systemic inflammation in thrombus formation. This is consistent with previous findings that neutrophil-mediated inflammation contributes to endothelial injury and thrombosis (23, 24). In the present study, WBC, NEU, and NEU% in the PE group were higher than those in the control group, and an elevated NEU was independently associated with PE. Lower LYM% may be related to lymphocyte apoptosis during systemic inflammation (25). During acute inflammatory responses, levels of CRP and FER increase; this response is regulated by a network of inflammatory mediators, including interleukin (IL)-6 and tumor necrosis factor (TNF). In a study by Hou et al. (26), IL-6 levels were higher in children with MPP complicated by PE than in those without PE, suggesting that elevated IL-6 may predispose patients to PE. In contrast, Zhang et al. (13) reported no significant differences in IL-6 levels between the PE and non-PE groups in MPP. In the present study, IL-6 levels were numerically higher in the PE group than in the control group, but the difference was not statistically significant. Because IL-6 was measured in only 13 patients in the PE group and 13 patients in the control group, this result should not be interpreted as evidence against a role of IL-6 in PE. Owing to the limited number of available IL-6 measurements, IL-6 was not included in the multivariable logistic regression model. The independent association between ESR and PE is also consistent with a previous report (27).

In the present study, co-pathogen infection was less common in the PE group than in the control group. This finding appears counterintuitive because PE generally reflects more severe disease. One possible explanation is that the control patients were selected across a broad study period from October 2019 to March 2025, during which the seasonal and annual prevalence of bacterial and viral co-pathogens may have varied. Therefore, differences in the temporal distribution of selected controls may have influenced the observed rate of co-pathogen detection. Another possibility is that M. pneumoniae-dominant infection may be sufficient to trigger a strong inflammatory and prothrombotic response in some children. Because of the retrospective design, this finding should be interpreted cautiously and requires confirmation in future studies with time-matched controls.

In the present study, D-dimer had the largest AUC (0.806), indicating good discriminative ability for PE. Although the seven-variable multivariable model showed good apparent discrimination, it was fitted primarily to estimate adjusted associations rather than to develop a clinical prediction tool. Bootstrap internal validation yielded an apparent AUC of 0.886 and an optimism-corrected AUC of 0.865. These performance estimates should nevertheless be considered exploratory because they were derived from the same single-center dataset and require external validation. Compared with previous studies, the risk factors identified in this study were broadly consistent (13, 15). Gu et al. (28) reported that, among children with MPP who underwent CTPA because of suspected PE, age, D-dimer level, and bilateral lung consolidation were independent predictors of PE. Similarly, the present study found that D-dimer and severe pulmonary involvement were associated with PE, supporting the clinical relevance of coagulation activation and extensive lung inflammation. However, the present study differed from Gu et al. in several respects. We included a larger number of PE cases and focused on children with severe MPP, whereas Gu et al. included children with MPP who underwent CTPA for suspected PE. In addition, our multivariable analysis identified prolonged fever, extrapulmonary symptoms, neutrophil count, ESR, APTT, D-dimer, and cardiovascular complications as factors independently associated with PE, while age and bilateral lung consolidation were not retained as final independent factors. These differences may be related to differences in study population, disease severity, candidate variables, and model construction.

Currently, the imaging diagnosis of pediatric PE primarily relies on CTPA and CTA (29). Previous studies on pediatric MPP complicated by PE have found that emboli are primarily located in the bilateral lower lobes, predominantly in the right lower lobe (30, 31), and the likelihood of PE increases with the number of lobes involved by infection (28), which is consistent with the findings of the present study. In this study, based on the spatial relationship between thrombi and pulmonary consolidation or atelectasis on imaging, only 20% of PEs were confined to pulmonary consolidation or atelectasis areas, whereas 64% occurred outside these areas and 16% involved both inside and outside regions, highlighting the need to examine pulmonary vessels beyond visibly infected areas. All patients in the PE group underwent vascular ultrasonography of the extremities after PE was confirmed, and only seven had concomitant peripheral vascular thrombosis of the extremities. Together, these findings suggest that PE in children with SMPP may differ from classic adult PE caused by embolic migration from lower-limb deep venous thrombosis. The distribution of thrombi does not support a purely local extension of inflammation from adjacent infected lung tissue. Instead, severe inflammation, recurrent high fever, reduced fluid intake, systemic hypercoagulability, and multifocal inflammatory vascular injury may all contribute to thrombus formation. Therefore, PE in children with SMPP may involve both *in situ* pulmonary vascular thrombosis and systemic thromboembolic mechanisms. When pulmonary vessels are embolized, local ischemia occurs, leading to necrosis and infarction of surrounding lung tissue. A study of 14 pediatric cases of MPP with PE also found discrepancies between the locations of emboli and those of necrotizing pneumonia, suggesting that multiple vascular thrombotic occlusions may occur beyond large vessels (32). PE can cause ischemic pleuritis, which may lead to a pleural effusion (8).

Early prevention of PE, in addition to optimizing anti-infective therapy, performing bronchoscopy, and pleural puncture to reduce inflammation, should include limb mobilization, local massage, and adequate hydration (10). Once PE occurs, management includes supportive care and anticoagulation, which remains the cornerstone of treatment. LMWH is generally preferred in pediatric patients due to its lower bleeding risk and ease of use (33). The 2018 American Society of Hematology guidelines for the management of venous thromboembolism recommend LMWH for ≤3 months of anticoagulation with risk factors, while LMWH should be administered for 6–12 months in cases without risk factors (34). Oral anticoagulants, such as warfarin, may be used with appropriate INR monitoring (9), while direct oral anticoagulants have also been increasingly applied in recent years. In this study, the initial anticoagulation choice for children with PE was unfractionated heparin via intravenous bolus or continuous infusion, followed sequentially by warfarin or rivaroxaban. Later, the initial therapy was switched to subcutaneous LMWH, followed by warfarin or rivaroxaban. In this study, no PE-related deaths occurred, and anticoagulation therapy was effective, with no bleeding events observed during follow-up. Thrombi resolved in 34% of patients within 1 month and in 82% within 3 months. This favorable prognosis may be related to early recognition after CTPA/CTA examination, timely anticoagulation, relatively limited thrombotic burden in most patients, preserved cardiopulmonary reserve in children, or other pediatric-specific characteristics of PE. However, because right ventricular function and imaging-based severity markers were not systematically assessed, these explanations remain speculative. Thrombolytic therapy is not routinely recommended but may be considered in high-risk cases (35, 36). Catheter-directed or surgical interventions are reserved for selected severe patients (37, 38). Because catheter-directed thrombolysis uses a lower drug dose locally, the bleeding risk is comparatively lower than with systemic thrombolysis (14, 39).

This study has several limitations. First, the sample size was small, and the study was a single-center retrospective design. Multicenter studies with larger sample sizes are needed to further validate the findings. Second, the controls consisted of patients with SMPP who underwent CTPA or CTA and had negative imaging results for PE, which may introduce some selection bias. However, in our center, contrast-enhanced chest CT was not performed solely because of suspected PE; it was also used to assess pulmonary ischemia or necrosis, guide prognosis evaluation and follow-up planning, and identify severe complications in children with SMPP. Therefore, although selection bias may have influenced the estimated ORs to some extent, not all control patients were selected only because of PE-like clinical features. Third, some patients had incomplete laboratory or pathogen-testing data, limiting the effectiveness of certain analyses and potentially constraining the understanding of PE. Only 37 of 199 patients underwent macrolide resistance testing, partly because resistance testing was less commonly performed during the earlier study period when first-line macrolide therapy remained clinically effective. Therefore, the relationship between macrolide resistance and PE risk could not be meaningfully assessed. In particular, IL-6 was measured in only 13 patients in the PE group and 13 patients in the control group; therefore, the analysis of IL-6 was underpowered, and missing data bias could not be excluded. Because of the small number of IL-6 measurements, multiple imputation or sensitivity analysis was not considered reliable, and IL-6 was not included in the multivariable model. Fourth, this study primarily explored factors associated with PE rather than developing a clinical prediction model. Bootstrap internal validation with 1, 000 resamples yielded an apparent AUC of 0.886 and an optimism-corrected AUC of 0.865. The Hosmer–Lemeshow test and calibration plot showed no clear evidence of poor calibration. Nevertheless, these assessments were performed using the same single-center dataset, and external validation was not conducted. In addition, because LASSO was applied only to the seven variables retained in the multivariable model, it represented a penalized sensitivity analysis rather than an independent validation of variable selection. The model-performance results should therefore be interpreted as exploratory. Fifth, the anticoagulation strategy changed over the long study period, with a gradual shift from unfractionated heparin to LMWH and subsequent oral anticoagulants. Because outcomes were not stratified by anticoagulation regimen, treatment heterogeneity may have influenced thrombus resolution time and the interpretation of prognosis. In addition, this study did not systematically evaluate right ventricular involvement or perform imaging-based risk stratification using CTPA indicators such as right ventricular dilation, the right-to-left ventricular diameter ratio, interventricular septal position, or other markers of right ventricular pressure overload. Therefore, the relationship between thrombotic burden, right ventricular function, disease severity, and prognosis could not be fully assessed. Also, the pathogenesis of PE in the context of MPP remains unclear. Finally, this study was not designed to develop a clinical prediction model. Due to the limited sample size and number of PE events, the multivariable association model was not intended for clinical prediction. Future multicenter studies with larger sample sizes are needed to develop and internally and externally validate prediction models for PE in children with SMPP. Further studies combining clinical data with animal experiments are also needed to elucidate the underlying mechanisms.

## Conclusion

The early clinical symptoms of severe MPP complicated by PE are often subtle and easily missed. Fever duration, NEU, ESR, D-dimer, APTT, extrapulmonary symptoms, and cardiovascular complications are independently associated with PE in pediatric patients with severe MPP. Clinicians should be vigilant when children with severe MPP have prolonged fever, markedly elevated inflammatory markers and D-dimer, and concomitant cardiovascular complications. Early chest imaging should be performed to detect PE, allowing timely diagnosis and intervention to improve prognosis.

## Data Availability

The original contributions presented in the study are included in the article/**Supplementary Material**. Further inquiries can be directed to the corresponding authors.

## Supplementary material

Supplementary Figure 1 LASSO coefficient profiles of the seven variables retained in the multivariable logistic regression model.

Supplementary Figure 2 Calibration plot of the seven-variable multivariable logistic regression model.
