# Supplementary material for: Factors associated with pulmonary embolism in children with severe Mycoplasma pneumoniae pneumonia
Source: Front Immunol. 2026 Oct 9;17:1900602. doi: 10.3389/fimmu.2026.1900602 (PMC13690517; doi:10.3389/fimmu.2026.1900602)
Supplement: Supplementary file 1 [file Table1.docx]

**Table S1. Distribution of pulmonary embolism (n=100).**

| **Embolic distribution** |  | **% (n)** |
| --- | --- | --- |
| **Pulmonary involvement status** |  |  |
|  | Left lung | 28% (28) |
|  | Right lung | 41% (41) |
|  | Bilateral | 31% (31) |
| **Lung lobes involved** |  |  |
|  | Superior lobe of the left lung | 18% (18) |
|  | Inferior lobe of the left lung | 52% (52) |
|  | Superior lobe of the right lung | 17% (17) |
|  | Middle lobe of the right lung | 11% (11) |
|  | Inferior lobe of the right lung | 61% (61) |
| **Relationship between embolism and pulmonary consolidation / atelectasis** |  |  |
|  | Pulmonary consolidation / atelectasis area | 20% (20) |
|  | Pulmonary consolidation / atelectasis external | 64% (64) |
|  | Pulmonary consolidation / atelectasis inside and outside | 16% (16) |
| **Pulmonary embolism vessel** | Pulmonary artery | 92% (92) |
|  | Pulmonary vein | 3% (3) |
|  | Pulmonary artery, pulmonary vein | 5% (5) |

**Table S2.** ROC curve analysis results.

| Variables | Sensitivity | Specificity | AUC | Cutoff value | P-value | 95%CI |
| --- | --- | --- | --- | --- | --- | --- |
| D-dimer | 0.750 | 0.740 | 0.806 | 3.2 | <0.001 | 0.745-0.867 |
| N | 0.463 | 0.822 | 0.671 | 8.83 | <0.001 | 0.594-0.748 |
| ESR | 0.292 | 0.958 | 0.600 | 57.5 | 0.017 | 0.519-0.681 |

AUC: area under the curve; CI: confidence interval; N: neutrophils; ESR: erythrocyte sedimentation rate.
